# Strength vs. endurance in myotome assessment—a case for (further studies on) repeated measurements

**DOI:** 10.3389/fresc.2026.1670617

**Published:** 2026-03-13

**Authors:** Alexia Coulombe-Lévêque, Nicolas Dehors, René Pelletier, François Cabana, Jean-Pierre Dumas, Guillaume Léonard

**Affiliations:** 1 Research Center on Aging, Centre Intégré Universitaire de Santé et de Services Sociaux de L'Estrie - Centre Hospitalier Universitaire de Sherbrooke (CIUSSS de L’Estrie - CHUS), Sherbrooke, QC, Canada; 2 Faculty of Medicine and Health Sciences, Université de Sherbrooke, Sherbrooke, QC, Canada; 3 Faculty of Sciences, Université du Québec à Montréal, Montréal, QC, Canada

**Keywords:** fatigue, low-back pain, lumbar radiculopathy, muscle endurance, muscle strength, myotome assessment, physiotherapy, weakness

## Abstract

Myotome testing is often used to detect radiculopathy in patients with low back pain and radiating leg pain. While myotomes are typically assessed by testing the strength of maximum voluntary contraction, some authors suggest endurance should also be assessed, through sustained and/or repeated contractions. Repeated (*n* = 7) strength tests of the big toe extensor were conducted in two patients with confirmed L5 radiculopathy. Rank-sum tests were conducted for each participant to determine whether their affected leg was weaker. Weakness was observed in both patients with radiculopathy (P1 & P2); however, while weakness was apparent from the first trial in participant P1, it only became detectable after repeated trials in participant P2. Our results suggest that myotome assessment could, in some cases, benefit from repeated strength trials, although further research is needed to confirm the robustness of this conclusion. Additional studies should be conducted in individuals with confirmed radiculopathy to further investigate whether some patients with no immediate weakness show a strength deficit after repeated trials.

## Introduction

Lumbar radiculopathy is a common reason for consultation with healthcare professionals (1). Radiculopathy involves the compression of the nerve root, which can affect sensory and motor function (2). The gold standard for its diagnosis relies on imaging and needle electromyography, which can be costly, invasive, and plagued by long waitlists (1, 3, 4). Therefore, health professionals also rely on physical assessment, including neuromuscular examination—which typically involves testing reflexes, dermatomes, and myotomes (3–10)—as an initial screening step to detect the presence of radiculopathy (3, 4, 6, 9).

Despite its widespread use, the diagnostic accuracy of neuromuscular examination remains uncertain (1, 4, 6–8, 11). A systematic review assessing the diagnostic performance of neurological tests in detecting radiculopathy found that motor tests had poor to moderate sensitivity compared to magnetic resonance imaging (MRI) findings (11). However, the types of motor tests employed in the studies included in the review varied significantly, encompassing both functional and isometric strength assessment; among these, myotome weakness appears to be the most specific indicator of lumbosacral radiculopathy (3, 11).

Myotomes are commonly assessed by measuring the *strength* of a maximal voluntary contraction in certain key muscles associated with specific nerve roots, aiming to detect weakness in the affected limb relative to the unaffected limb (4, 6, 7, 9). Some clinical textbooks have suggested that muscle endurance [assessed by holding a contraction at least 5 s and/or performing it repeatedly (5, 8)] should be tested as part of the myotomal assessment, rather than relying solely on strength, but these recommendations do not appear to be based on scientific studies and are not widely adopted. To date, there seems to be no scientific literature specifically evaluating whether muscle strength or endurance is a more accurate indicator of the presence of radiculopathy. The lack of studies comparing these different indicators, combined with the uncertainty surrounding optimal assessment approaches, can lead to significant variability in both education and clinical practice.

## Methods

To gain a better understanding of neuromuscular *weakness* vs. *fatigue* as detectable signs of radiculopathy, we performed repeated strength assessments of the *extensor hallucis longus* (a muscle innervated by the L5 nerve root, responsible for big toe extension) in two patients (P1, male, 53 yo; & P2, male, 71 yo; patient characteristics are detailed in Table 1) with stable L5 radiculopathy. The radiculopathy was diagnosed by an orthopaedic surgeon (FC) using electromyography, who also confirmed the absence of central and peripheral neurological diseases. Both patients had consulted a physician for low back pain and referred pain in their dominant limb (though for P1, pain had resolved by the time of data collection). Neither patient presented hallux deformity, history of hallux pain, or had their limb immobilized in the past year. The study protocol was approved by the institutional ethics board of the CIUSSS de l'Estrie-CHUS, and verbal and written consent was provided by both patients.

**Table 1:** Patients sociodemographic and clinical characteristics.

Characteristics	Patient 1	Patient 2
Age (yo)	53	71
Dominance	R	R
Weight (lbs)	205	174
Height (cm)	178	183
Other health conditions	Diabetes type II, hypertension, bladder cancer (surgically removed)	Hemochromatosis, melanoma, chronic lymphocytic leukemia
Medication	metformin, perindopril	pregabalin, acetaminophen, celecoxib, clotrimazole, atorvastatin, acetaminophen + codeine, ciprofloxacin, dutasteride, solifenacin, trazodone, venlafaxine
Pain	None	Lower back and lateral side of right lower limb
Rolland-Morris	2	12
Brief Pain Inventory—Severity (0–40)	0	21
Brief Pain Inventory—Interference (0–70)	0	41

Both patients performed practice trials with submaximal contractions to familiarize themselves with the testing procedure. The formal assessment consisted of 7 contractions at maximum voluntary strength performed in a standardized position (120° plantar flexion). For each contraction, the highest strength rating recorded by the dynamometer (Chatillon™) was retained. A statistical analysis was conducted separately for each patient, to determine whether the average strength (i.e., the average of all 7 measurements) in the affected limb was significantly lower than the average strength in the healthy limb. On the recommendations of a biostatistician, this analysis was conducted using a rank-sum test, with *α* = .005 (12). This choice was informed by the fact that the observations are not paired (the n^th^ measure from one limb is not inherently related to the n^th^ measure from the other limb). Moreover, conducting a separate analysis for each participant (instead of pooling results) ensured that the postulate of independence was not violated.

## Results

**Figure 1:**
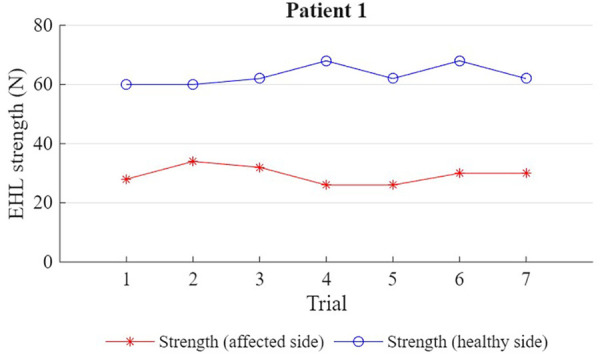
Shows the strength (N) of the EHL (extensor hallucis longus) muscle in the affected and unaffected limb for patient 1 (7 trials).

**Figure 2:**
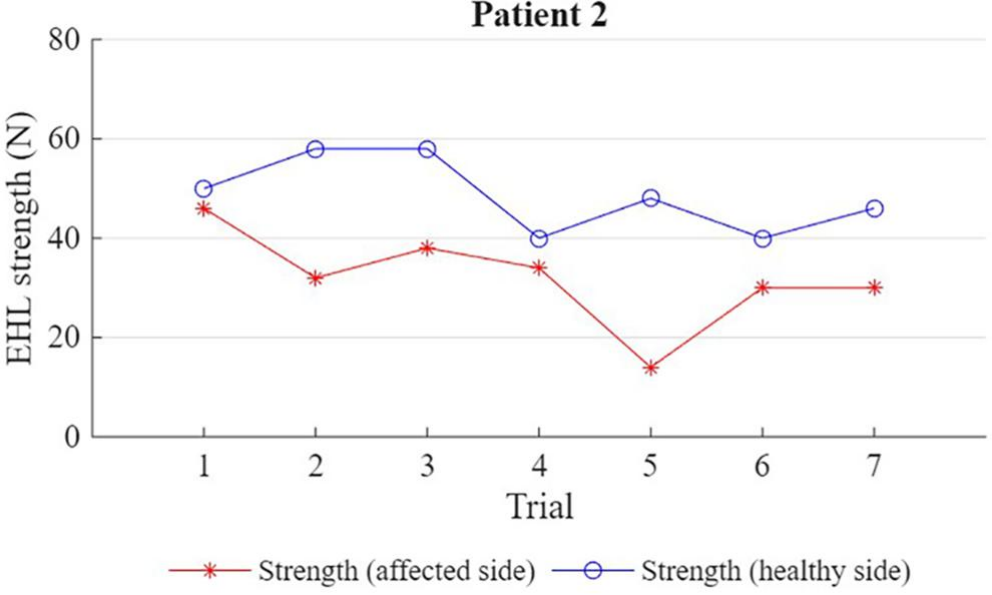
Shows the strength (N) of the EHL (extensor hallucis longus) muscle in the affected and unaffected limb for patient 2 (7 trials).

For both participants, 7 maximal strength measurements were obtained for the right and left hallux extensor muscle (Figures 1, 2). For P1, the affected limb was, on average, roughly half as strong as the healthy limb (29.4 ± 3.0 N vs. 63.1 ± 3.4 N; *p* = .001). For P2, the affected limb was, on average, roughly two thirds as strong as the healthy limb (32 ± 9.7 N vs. 48.6 ± 7.5 N; *p* = .002). However, while this difference in strength was apparent from the first trial in P1 (28 N vs. 60 N), P2's first trial yielded a similar strength score for both limbs (46 N vs. 50 N).

## Discussion

A neuromuscular assessment consisting of 7 maximum voluntary contractions of the hallux extensor muscle was performed in 2 individuals with a confirmed L5 radiculopathy. The repeated strength tests were used to assess the presence of neuromuscular *weakness* (an immediate and sustained decrease in strength score in the affected limb compared to the healthy limb) and neuromuscular *fatigue* (a gradual decrease in strength scores in the affected limb over multiple repetitions).

Both patients had a significantly lower average strength in the affected limb compared to the healthy limb. However, while weakness was immediately apparent in P1, P2's first contraction in the affected limb was as strong as in the healthy limb; it is only after repeated measures that a decrease in average strength could be detected.

P2's results could be a manifestation of *fatigue* (wherein the contraction was initially strong but weakened throughout the repeated trials, gradually bringing the average down) (5), or could simply be a result of high variability in the patient's strength scores (wherein *weakness* was present, but initially masked by a randomly high first trial and only apparent after multiple trials).

It is unclear why the two patients showed different strength patterns. Beyond normal inter-individual variation, it is possible that the difference between them was attributable to some clinical characteristic (medication, health condition, etc.) though we cannot draw a causal conclusion. Interestingly, the pain-free patient (P1) had an immediately apparent strength deficit, while the patient with back and leg pain (P2) showed no significant strength deficit on the first trial. While the experimental contractions did not induce or increase pain in either patient, these results also suggest that the general presence of pain was not a main driving factor behind the observed strength deficits.

Additional studies should be conducted to shed light on the presence of weakness and fatigue during myotome assessment. For instance, it would be worthwhile to identify the optimal number (and possibly duration) of strength trials required to identify motor deficits, balancing sensitivity and specificity against time-efficiency. Moreover, a study with a larger sample size could statistically assess temporal patterns—i.e., the possible presence of fatigue across trials, which would be evidenced by a decrease in strength over time steeper in the affected limb compared to the healthy limb. A larger sample size would also allow for better control of possible confounding factors such as comorbid health conditions, the presence and intensity of pain, and medication usage. Including patients with involvement of different nerve roots would also increase the validity of the study, enhancing internal validity by allowing more robust conclusions about physiological mechanisms if consistent patterns emerge across different nerve roots, and improving external validity by making clinical recommendations applicable to a larger patient population. It is also possible that different results may be obtained for different muscle groups affected. Notably, it is plausible that big toe extension, which is a somewhat awkward movement to perform, may yield more variable strength scores across trials compared to movements like elbow flexion or knee extension.

It remains unclear whether fatigue or variability was responsible for P2's high initial strength and subsequent decrease, and our limited sample size prevents us from drawing robust conclusions. However, our results do suggest that clinical testing for myotome involvement should use multiple measurements and look for a decrease in *average* strength in the affected limb, rather than drawing conclusions based on a single strength test. Illustrating this point, a recent study (13) conducted on 227 patients with cervical radiculopathy reported motor *weakness* in just under half of the sample—begging the question whether the remaining 117 patients might have shown some deficit had repeated strength trials been conducted. Myotome assessment procedures should be further investigated to optimize and standardize clinical screening tools.

In conclusion, our findings highlight the importance of additional research to validate and expand upon our observations, and to determine optimal procedures for myotome assessment. Until further evidence is available, we tentatively suggest that clinicians perform multiple strength assessments before ruling out a possible myotome deficit and potential radiculopathy.

## Data Availability

All original data analyzed in this study are included in the article; further inquiries can be directed to the corresponding author.
